# Clinical and molecular features of primary spinal epidural lymphomas

**DOI:** 10.1007/s00277-025-06554-0

**Published:** 2025-09-25

**Authors:** Louisa Adolph, Veit M. Stoecklein, Verena Passerini, Michael Heide, Philipp Karschnia, Stefan Zausinger, Louisa von Baumgarten, Michael von Bergwelt-Baildon, Jörg Christian Tonn, Sophia Stoecklein, Niklas Thon, Christian Schichor, Martina Rudelius, Oliver Weigert

**Affiliations:** 1 Department of Internal Medicine III, Laboratory for Experimental Leukemia and Lymphoma Research (ELLF), Ludwig-Maximilians University (LMU) Hospital, Max-Lebsche-Platz 30, Munich, Germany; 2 Department of Neurosurgery, Ludwig-Maximilians University (LMU) Hospital, Munich, Germany; 3 German Cancer Consortium (DKTK), German Cancer Research Center (DKFZ), Munich, Heidelberg, Germany; 4 Department of Neurosurgery, Friedrich-Alexander-University (FAU) Hospital, Erlangen-Nuremberg, Germany; 5 Department of Neurology, Ludwig-Maximilians University (LMU) Hospital, Munich, Germany; 6 Department of Radiology, Ludwig-Maximilians University (LMU) Hospital, Munich, Germany; 7 Institute of Pathology, Ludwig-Maximilians University (LMU), Munich, Germany

**Keywords:** Lymphoma, Primary spinal epidural lymphoma, Clinical aspects, Molecular analysis, Pathology

## Abstract

Lymphomas confined to the spinal epidural space at initial diagnosis are rare and referred to as primary spinal epidural lymphoma (PSEL). This study presents clinical, histopathological, and molecular insights from 13 PSEL cases, including follicular lymphoma (PSEL-FL, *N* = 4), extranodal marginal zone lymphoma (PSEL-MZL, *N* = 3), diffuse large B-cell lymphoma (PSEL-DLBCL, *N* = 5), and Burkitt lymphoma (PSEL-BL, *N* = 1). Despite localized presentation at initial diagnosis, PSEL with aggressive histologies demonstrated remarkably unfavorable outcomes, contrasting the highly indolent clinical course observed in indolent PSEL, particularly PSEL-FL. Molecular analysis showed similarities to nodal counterparts and separation by entity. Comparison of PSEL-FL and classic FL revealed activated tumor microenvironment-associated pathways, potentially supporting extranodal lymphoma growth in this rarely affected spinal epidural site.

**Supplementary Information:**

The online version contains supplementary material available at 10.1007/s00277-025-06554-0.

## Introduction

10% of all spinal epidural tumors are malignant lymphomas [1]. However, only 0.1–3.3% of all patients with non-Hodgkin lymphoma (NHL) have spinal epidural manifestations [2–5]. Lymphomas confined to the spinal epidural space at initial diagnosis are even rarer and referred to as primary spinal epidural lymphoma (PSEL) [6]. Only a few retrospective case reports and case studies have been published. The largest retrospective case series reported clinical data on 52 patients with PSEL treated at institutions of the Rare Cancer Network. In here, PSEL was mainly diagnosed at an older age with a male predominance. The thoracic spine was the most common disease site with motor weakness and back pain being the most common initial symptoms [6]. Due to the rarity of the disease, the clinical and molecular characteristics of these lymphomas are not well defined.

PSEL is not restricted to a particular lymphoma entity, but can present with various histologies, with aggressive B cell NHL reported as the most prevalent [2, 6]. The cellular origin and pathogenesis of PSEL remain unclear. It may result from the malignant transformation of lymphoid cells in the epidural space [7] or from precursor cells in paraspinal tissues or blood that migrate to the spinal epidural space [8, 9]. To our knowledge, no studies have examined the molecular characteristics that may explain the unique clinical presentation of PSEL. Here, we report a comprehensive analysis of clinical, histopathological and molecular features in a cohort of strictly defined PSEL.

## Materials and methods

We searched the electronic medical record database of the LMU Hospital covering the years 2003 to 2019 for patients with “lymphoma” and “spinal involvement”. We excluded cases with systemic or other extraspinal manifestations. Diagnosis of PSEL was confirmed by expert histopathological review in all cases. Written informed consent had been obtained from all patients prior to surgery, which included the use of medical data for scientific purposes. All cases with available formalin-fixed and paraffin-embedded (FFPE) material were used for further analyses. All study-related procedures, including the retrieval of archived biopsy material and all molecular analyses were approved by the local ethics review board of the Ludwig-Maximilians-University (LMU ID #20–176). A detailed description of procedures, data analysis and statistics is provided in the supplement.

## Results

**Fig. 1:**
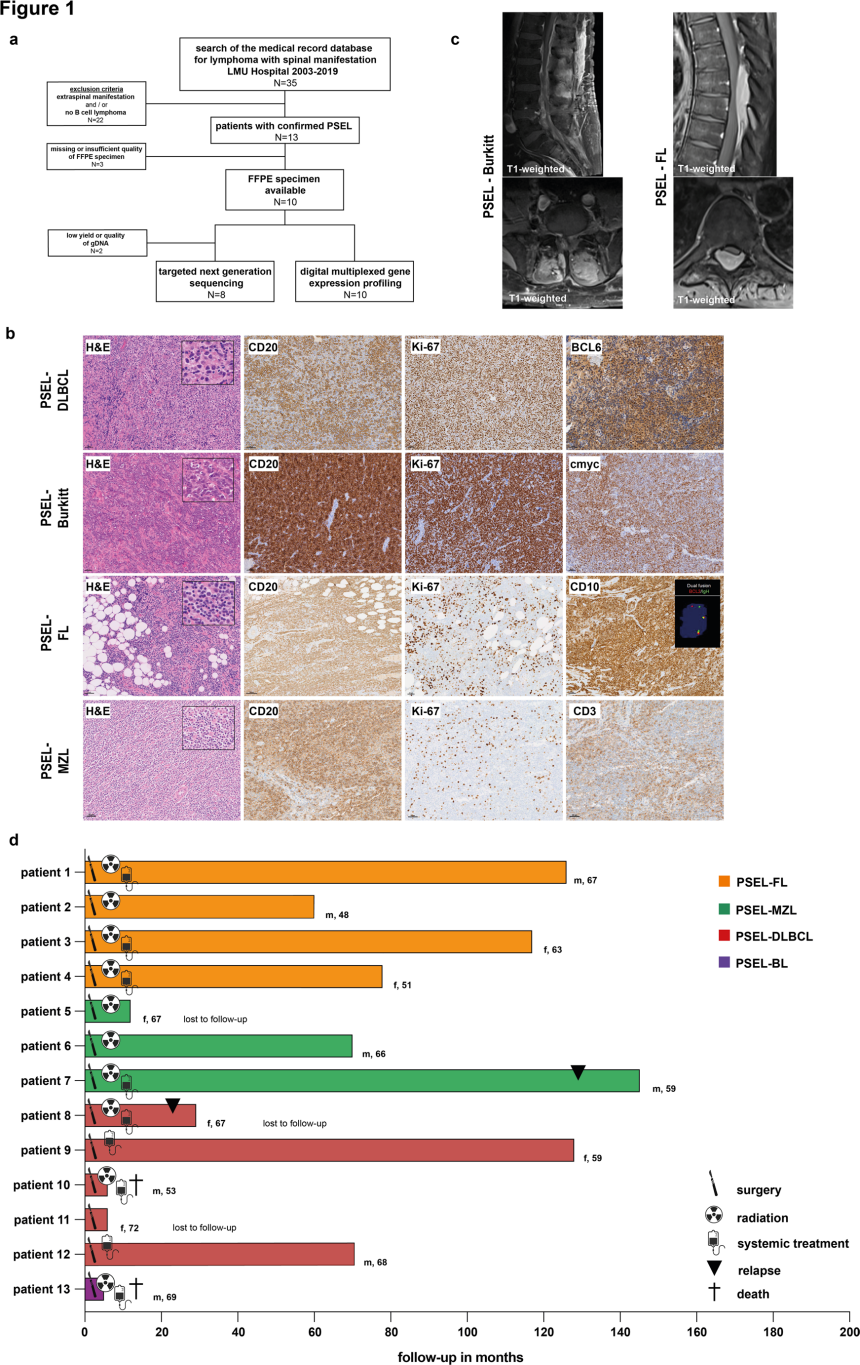
Immunohistochemical and clinical characteristics of the PSEL cohort. **a** CONSORT diagram of the study. **b** H&E stains, immunohistochemistry for CD20, Ki-67, BCL6, cmyc, CD10 and CD3. FISH dual fusion IgH/BCL2 (inlet). **c** Sagittal and transverse plane MRI scan of the spine, T1-weighted. **d** Swimmers plot of PSEL cohort. Treatment, relapse and death are indicated accordingly

The search of our medical record database for lymphoma with spinal manifestation identified 35 cases. We excluded cases with additional extraspinal manifestations (*N* = 18) and/or histologies other than B cell lymphoma (*N* = 4; thereof two cases of Ewing sarcoma and two cases of multiple myeloma, respectively). In total we identified 13 patients with PSEL (Fig. 1a). The median age at diagnosis was 66 years (range 48–72 years). Seven patients were male and six female with equal distribution among the lymphoma entities. Of the 10 cases evaluable for clinical presentation prior to surgery, eight patients (80%) experienced pain, and six patients (60%) had neurological deficits, mostly hypaesthesia. Patient and disease characteristics are summarized in Table S1. All patients underwent decompressive laminectomy with resection of the epidural mass.

### Histopathology

Histopathological analysis showed aggressive NHL in six cases, specifically diffuse large B cell lymphoma (PSEL-DLBCL) in five cases and Burkitt lymphoma (PSEL-BL) in one case. All PSEL-DLBCLs were characterized by diffuse infiltration of polymorphic lymphoblasts, prominent angiogenesis, and identified as germinal center B-cell (GCB)-like subtype by the Hans classifier [10]. In contrast to published results [6], indolent NHLs were as prevalent as aggressive NHLs in our series (7/13, 53%), including four cases of follicular lymphoma (PSEL-FL) and three cases of extranodal marginal zone lymphoma (PSEL-MZL) (Fig. 1b). PSEL-MZLs resembled their nodal counterpart. Interestingly, PSEL-FLs predominantly showed a diffuse growth pattern.

### Imaging

PSEL were located in the cervical spine (*N* = 1), thoracic spine (*N* = 7), lumbar spine (*N* = 4), and S1/S2 (*N* = 1) (Fig. 1c). We re-evaluated all available pre-surgical MRIs (*N* = 8). Assessment of tumor sizes by sagittal T2-weighted images showed no differences between aggressive and indolent PSEL (Table S1). All tumors showed epidural masses with homogeneous contrast enhancement on T1-weighted images. Tumor signal intensity was standardized to cerebrospinal fluid (CSF), showing a trend toward a higher signal intensity/CSF ratio in aggressive versus indolent PSEL, though not statistically significant, likely due to the small sample size (Fig. S1a).

### Postsurgical treatment and patient outcome

The clinical course of all patients is summarized in Fig. 1d and Table S1. Median follow-up was 70 months (range 5–145 months). 10 patients (77%) received adjuvant radiotherapy (21.6–40 Gy), including all with indolent PSEL. Systemic treatment followed GMALL B-NHL/B-ALL consensus guidelines for PSEL-BL, and R-CHOP or R-CHOEP for 4 of 5 PSEL-DLBCL patients (83%). Patients with indolent PSEL receiving systemic treatment were primarily treated with rituximab monotherapy (43%), except for one PSEL-FL patient who received R-CHOP. The CNS-IPI indicated low, intermediate, and high risk in one (17%), three (50%), and one (17%) patients with aggressive PSEL, respectively. CSF examination, performed in 10 of 13 patients, revealed pleocytosis in three cases (30%), with suspected meningeosis lymphomatosa in one (PSEL-DLBCL, patient no. 12) and confirmed meningeosis lymphomatosa in another (PSEL-BL, patient no. 13). Two intermediate-risk PSEL-DLBCL patients received CNS-directed therapy with high-dose methotrexate or DHAP and intrathecal methotrexate, and one also received intrathecal methotrexate prophylaxis. The PSEL-BL patient with confirmed meningeosis lymphomatosa received CNS-directed therapy with high-dose and intrathecal methotrexate.

The patient with PSEL-BL died from a CNS relapse during initial therapy, 5 months postoperatively. Another PSEL-DLBCL patient (patient no. 10) died 6 months postoperatively from a CNS relapse, with an intermediate CNS-IPI risk, normal CSF, and no prophylactic CNS treatment. In contrast, a second PSEL-DLBCL patient (patient no. 12) with intermediate CNS-IPI risk and initial pleocytosis, who received prophylactic CNS treatment, achieved complete remission and remains in remission after 71 months. Another PSEL-DLBCL patient (patient no. 8) relapsed with multifocal disease 23 months after diagnosis. Overall, the outcome for aggressive NHL patients in our cohort is remarkably poor, with two deaths (33%), one relapse (17%), and only two long-term responders (33%) after a median follow-up of 99.5 months.

In contrast, there were no reported deaths in the subgroup of PSELs with indolent histologies. Only one patient with PSEL-MZL (patient no. 7) showed systemic disease relapse with lymphadenopathy, bone marrow infiltration and monoclonal gammopathy 129 months after surgery, while all other patients with indolent PSEL remained in ongoing complete remission. Not a single patient with PSEL-FL relapsed after a median follow-up period of 97.5 months in this cohort (Fig. 1d, Table S1).

### Targeted mutational landscape

**Fig. 2:**
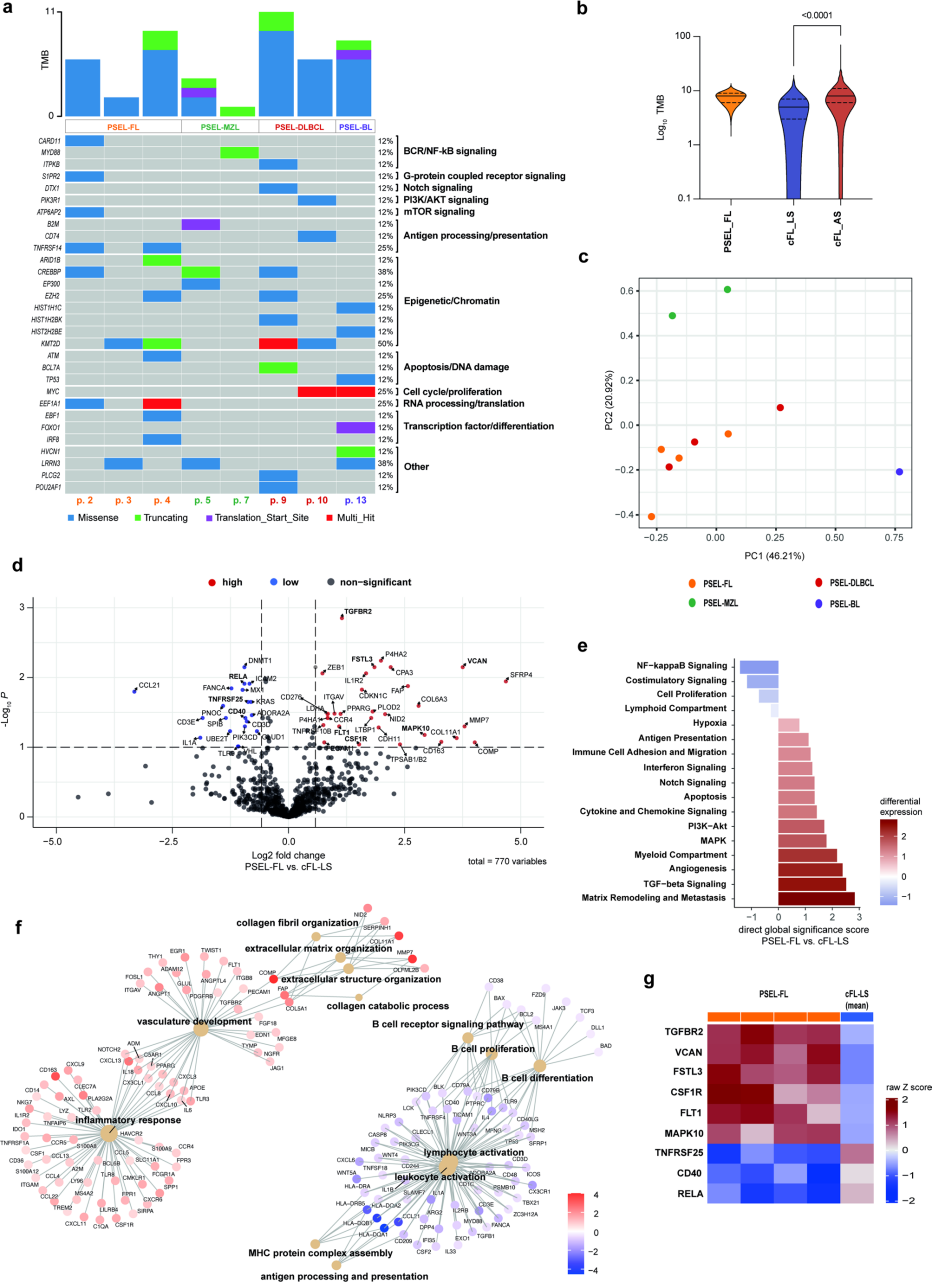
Targeted sequencing and digital multiplexed gene expression analysis. **a** Oncoplot of non-silent variants with a VAF > 10%. **b** Comparison of mean TMB (number of non-silent variants (VAF > 10%) per sample) between PSEL-FL, cFL-LS and cFL-AS cohort. **c** PCA of top one hundred differentially expressed genes comparing PSEL entities (IO360 panel, Nanostring). **d** Differential gene expression comparing PSEL-FL versus cFL-LS (IO360 panel, Nanostring). **e** Global significance scores comparing PSEL-FL and cFL-LS. **f** Cnetplot showing individual genes associated with selected enriched GO gene sets comparing PSEL-FL and cFL-LS. **f** Heatmap of selected differentially expressed genes comparing PSEL-FL with mean expression in cFL-LS

The targeted mutational profiles of all evaluable PSEL are depicted in Fig. 2a and Table S2. Largely, the mutational profile of PSEL reflected the mutational landscape of their nodal counterparts (Fig. S1b) [11]. One patient with GCB-like DLBCL showed mutations in *EZH2*, *KMT2D* and *CREBBP* most consistent with the EZB/C3 subtype in nodal DLBCL [12]; this patient was in ongoing complete remission after treatment with R-CHOP at last follow-up (128 months). The other evaluable patient with PSEL-DLBCL showed a multi-hit *MYC* mutation (but no *MYC*- or *BCL2*-translocation) and a *KMT2D* mutation. This patient had early CNS relapse and died during salvage therapy.

Previous analyses have reported distinct mutational profiles of extranodal MZL from various origins but did not include primary epidural localization [13]. One PSEL-MZL case had mutations in the acetyltransferase genes *CREBBP* and *EP300*, commonly found in a variety of extranodal MZL, while the other case showed only a *MYD88* mutation. Notably, *MYD88* mutations have been less commonly reported in MZL, mostly in orbital MZL [13]. Our PSEL-MZL exhibited a *MYD88* L265P mutation, known to constitutively activate NF-κB signaling and associated with extranodal disease and inferior outcomes in various B cell lymphomas [14]. Interestingly, this case (patient no. 7) was the only PSEL with indolent morphology that subsequently progressed to systemic disease.

Genes known to be recurrently mutated in classic FL (cFL) were also found in the PSEL-FL cohort, including the epigenetic modifiers *KMT2D*, *CREBBP*, and *EZH2*, the mTOR gene *ATP6AP2*, as well as signaling molecules *TNFRSF14* and *CARD11*. All cases lacked *STAT6* mutations. Interestingly, mutations in *EEF1A1* have been reported in only 9% of cFL but were found mutated in 2 out of 4 PSEL-FL (50%) [15]. A higher mutation frequency was previously reported in duodenal-type FL (DTFL), another rare and highly indolent FL subtype [16]. *KMT2D* mutations in PSEL-FL were all heterocygous, as previously observed for DTFL and limited stage cFL. Since we and other have previously reported that there is an increasing frequency of multiple/biallelic *KMT2D* mutations during the progression of FL, this suggested that PSEL-FL is biologically more closely related to early stages of FL [16, 17]. Therefore, we next compared the targeted mutational burden (TMB) of PSEL-FL with available reference cohorts for limited stage cFL (cFL-LS) and advanced stage cFL (cFL-AS). Indeed, the TMB of PSEL-FL was lower compared to cFL-AS and more aligned with the range observed in cFL-LS (Fig. 2b). The targeted mutational profile of the PSEL-FL cohort was also comparable with the mutational profile of the cFL-LS reference cohort (Fig. S1c). Thus, the mutational landscape of PSEL-FL resembles that of early/limited stage disease, consistent with our observation of a highly indolent clinical course.

### Gene expression profiling

We used the nCounter IO360 panel to comprehensively analyze the expression of 770 curated immune-oncology genes in the PSEL cohort. Principal component analysis showed distinct clustering of PSEL-MZL and PSEL-BL, while PSEL-FL clustered with PSEL-DLBCL, aligning with their GCB-like subtype (Fig. 2c).

Recognizing that cFL relies heavily on the interactions with the tumor microenvironment (TME) of lymphoid tissues, we sought to identify distinct TME-related gene expression patterns that allow PSEL-FL to thrive in the epidural space. Differential gene expression analysis of PSEL-FL and its nodal counterpart cFL-LS identified significantly up-regulated (*N* = 20) and down-regulated (*N* = 32) genes; including lower expression of the T cell chemotactic cytokine *CCL21*, and higher expression of genes involved in the organization of extracellular matrix and angiogenesis, including *SFRP4*, *COMP*, *MMP7*, and *VCAN* (Fig. 2d, Table S3). To measure overall changes in pathway activities, we performed gene set analysis using direct global significance score and gene set enrichment analysis (GSEA). Indeed, this indicated activated matrix remodeling and angiogenesis/vascular development in PSEL-FL as compared to cFL-LS (*VCAN*, *FSTL3*, *FLT1*) (Fig. 2e, f,g). Furthermore, we found lower NF-κB- and co-stimulatory signaling (*CD40*, *RELA*, *TNFRSF25*) (Fig. 2e, g), as well as reduced B cell receptor (BCR) signaling, B cell proliferation and differentiation (Fig. 2f). Vice versa, we identified potentially compensatory activated inflammatory pathways, including TGF-beta and WNT, MAPK and PI3K-AKT signaling (*TGFBR2*, *MAPK10*, *CSF1R*, *FLT1*) (Fig. 2g). We confirmed our findings by multiplex immunfluorescence: PSEL-FLs were characterized by high vessel density (VEGFR2), elevated TGF-beta expression and increase in p-MAPK positive cells in the local microenvironment (Fig. S1d). These findings reveal transcriptional programs that may contribute to lymphoma growth in the spinal epidural space in PSEL-FL.

## Discussion

Here, we describe the distinct clinical and molecular features of PSEL. Aggressive PSEL exhibit unexpectedly poor outcomes despite being limited stage at initial diagnosis, with high rates of subsequent CNS involvement. In contrast, our data suggest that indolent PSEL may not be as rare as previously reported [6] and exhibit an exceptionally indolent clinical course, particularly PSEL-FL. Histopathological analysis of PSEL-FL showed a diffuse growth pattern, unlike the follicle-like pattern typically seen in classic follicular lymphoma (cFL), and instead resembling the diffuse variant of follicular lymphoma, a subtype of FL with uncommon features (uFL) as recognized in the updated WHO classification [18]. Notably, although not identical, the diffuse variant of uFL overlaps with a subentity recognized by the International Consensus Classification (ICC), namely *BCL2*-translocation-negative, CD23-positive follicular center lymphoma, many of which harbor *STAT6* mutations [19]. However, all of our PSEL-FL cases were positive for the t(14;18) translocation, negative for CD23, and did not harbor *STAT6* mutations. Therefore, we propose that PSEL-FL may represent a distinct subentity with a particularly indolent clinical course. Interestingly, gene expression profiles and multiplex immunofluorescence imaging revealed that PSEL-FLs exhibit distinct transcriptional programs, including increased expression of genes involved in extracellular matrix organization and angiogenesis, as well as inflammatory pathways driven by TGF-beta/MAPK rather than BCR/NF-κB. These features may potentially support extranodal lymphoma growth in this rarely affected epidural space.

In summary, despite the small cohort size and the inherent limitations in generalizability, our study provides new clinically and biologically relevant insights into this very rare group of patients presenting with PSEL.

## Supplementary Information

Below is the link to the electronic supplementary material.

Supplementary Material 1

(PNG 17.8 MB)

High Resolution Image (TIF 142 MB)

Supplementary Material 3

Supplementary Material 4

Supplementary Material 5

## Data Availability

Data is provided within the manuscript or supplementary information files. Omics data generated and analyzed during this study are available from the corresponding author upon reasonable request.
