# Supplementary material for: Clinical and molecular features of primary spinal epidural lymphomas
Source: Ann Hematol. 2025 Sep 25;104(10):5493–9. doi: 10.1007/s00277-025-06554-0 (PMC12619745; doi:10.1007/s00277-025-06554-0)
Supplement: Supplementary file 1 — Supplementary Material 1 [file 277_2025_6554_MOESM1_ESM.docx]

# Clinical and molecular features of primary spinal epidural lymphomas

# Supplement

**Journal:** Annals of Hematology

**Authors**: Louisa Adolph^1*^, Veit M. Stoecklein^2,3*^, Verena Passerini^1^, Michael Heide^1^, Philipp Karschnia^2,3,4^, Stefan Zausinger^2^, Louisa von Baumgarten^2,3,5^, Michael von Bergwelt-Baildon^1,3^, Jörg-Christian Tonn^2^, Sophia Stoecklein^6^, Niklas Thon^2,3^, Christian Schichor^2^, Martina Rudelius^7*^ and Oliver Weigert^1,3*^

*These authors contributed equally

**Affiliations**:

^1^ Department of Internal Medicine III, Laboratory for Experimental Leukemia and Lymphoma Research (ELLF), Ludwig-Maximilians University (LMU) Hospital, Munich, Germany.

^2^ Department of Neurosurgery, Ludwig-Maximilians University (LMU) Hospital, Munich, Germany.

^3^ German Cancer Consortium (DKTK), Munich, Germany, and German Cancer Research Center (DKFZ), Heidelberg, Germany.

^4^ Department of Neurosurgery, Friedrich-Alexander-University (FAU) Hospital, Erlangen-Nuremberg, Germany.

^5^ Department of Neurology, Ludwig-Maximilians University (LMU) Hospital, Munich, Germany

^6^ Department of Radiology. Ludwig-Maximilians University (LMU) Hospital, Munich, Germany.

^7^ Institute of Pathology, Ludwig-Maximilians University (LMU), Munich, Germany.

**Correspondence:**

Oliver Weigert, MD. Laboratory for Experimental Leukemia and Lymphoma Research, Department of Medicine III, Ludwig-Maximilians University (LMU) Hospital, Max-Lebsche-Platz 30, D-81377 Munich, Germany

Tel. +49 89 4400 43985 // Fax. +49 89 4400 43970

Email: oliver.weigert@med.uni-muenchen.de

**Supplemental Materials and Methods**

Immunohistochemistry and multispectral imaging analysis:

Diagnosis of PSEL was confirmed by expert histopathological review in all cases (M.R.). Immunohistochemistry and multispectral imaging analysis was performed as previously described [1]. 2 μm tissue sections were deparaffinized in xylene and rehydrated in graded alcohol and distilled water. Heat induced antigen retrieval was performed on tissue sections. Hematoxylin eosin staining was performed. The following antibodies were used: CD20 1:100 (mouse monoclonal [clone L26], Medac, Wedel, Germany), Ki-67 1:200 (rabbit monoclonal [clone SP6], Millipore-Sigma, Darmstadt, Germany ), BCL6 1:50 (mouse monoclonal [clone 124], Agilent Technologies, Waldbronn, Germany), cmyc 1:50 (rabbit polyclonal [clone EP121], Millipore-Sigma, Darmstadt, Germany ), CD10 ready to use (rabbit monoclonal [clone SP67], Roche Diagnostics, Mannheim, Germany).

Dual-color dual-fusion fluorescence in situ hybridization (FISH) analysis was performed for qualitative detection of the translocation t(14;18)(q32.3;q21.3)  in PSEL-FL using the ZytoLight® SPEC BCL2/IGH Dual Color Dual Fusion Probe (Zytomed) (N=4).

Multispectral imaging analysis was performed using a Vectra® Polaris System. 1.5 mm thick tissue slides from FFPE-blocks were prepared and incubated overnight in a drying oven at 50-55 °C, followed by deparaffinization in xylene two times for 15 min. Slides were rehydrated using an ethanol gradient (5 min ethanol absolute, 5 min 96% ethanol, 5 min 70% ethanol) ending with distilled water. Rehydrated slides were incubated in 4% buffered formalin for 20 min at room temperature and each slide was washed 3 times in distilled water for 2 min. Finally, slides were washed 3 times with 0.05% Tween-20 TBS buffer for 2 min each. Staining was performed with the Opal™ 7 Tumor Infiltrating Lymphocyte Kit (Cat. #OP7TL1001KT, Akoya Biosciences, Marlborough MA, USA) according to the manufacturers standard protocol. The following four antibodies were applied: CD20 1:100 (mouse monoclonal [clone L26], Medac, Wedel, Germany), VEGFR2 1:100 (rabbit monoclonal [clone 55B11], Cell Signaling Technologies, Danvers MA, USA), TGFß 1:100 (rabbit monoclonal [clone EPR21143], Abcam, Cambridge, UK), phospho-MAPK 1:200 (rabbit polyclonal [clone D13.14.4E], Cell Signaling Technologies, Danvers MA, USA). Pictures were taken using the quantitative slide scanner PhenoImager HT (Akoya Biosciences, Marlborough MA, USA) with the Vectra Polaris 1.0.7 and Phenochart 1.0.8 software.

Imaging:

Signal intensity of the tumor on T1- and T2-weighted MRI images was determined using Visage 7.1 software (Visage Imaging) and standardized to the signal intensity of CSF on the same scan as previously described [2].

Targeted DNA sequencing:

Genomic DNA (gDNA) and RNA were isolated from FFPE samples using the AllPrep DNA/RNA FFPE Kit (QIAGEN). We performed targeted DNA sequencing of recurrently mutated genes as previously described for all cases with sufficient yield and quality of gDNA (N=8) [3]. DNA was fragmented (Covaris sonication) to 250 bp and further purified using Agentcourt AMPure XP beads (Beckman Coulter). Size-selected DNA was then ligated to adaptors during library preparation. Libraries were enriched for genes using the Sure Select XT Target Enrichment System for Illumina Paired-End Multiplexed Sequencing and each capture pool was sequenced at 300-400*x.* Read pairs were aligned to the hg19 reference sequence using the Burrows-Wheeler Aligner [4], and data were sorted and duplicate-marked using Picard tools (version 2.23.3). All steps were performed within the bcbio-nextgen toolkit (version 1.2.4). The minimum quality criterion was 80% of target bases have > 30x sequencing coverage. Cases with 60-79% of target bases with > 30x sequencing coverage were also included if target bases not covered was < 1%. Cases with target bases covered 30x < 60% or cases with target bases covered 30x between 60-80% and target bases not covered > 1% were excluded. Metrics were collected using Picard tools (version 2.23.3). Mutation analysis for single nucleotide variants (SNV) and Insertions and Deletions (InDels) was performed using MuTect2 (GATK v4.1.9.0) and annotated by Funcotator (GATK v4.1.9.0). A panel-of-normals (PON) filter was generated from non-tumor samples collected previously (N=5). Tumor samples were filtered with PON and candidate variants were rejected as germline events or sequencing artefacts if present in two or more normal samples. Non-silent variants (Missense_Mutation, Truncating_Mutation, Translation_Start_Site, Multi_Hit) resulting from BestEffect Funcotator annotation (dataSources.v1.6) at a variant allele frequency (VAF) of > 10% were kept for further investigations. Germline polymorphisms and sequencing artefacts were excluded by comparison with the PON and with the gnomAD database. Known germline polymorphisms from the Exome Sequencing Project and dbSNP databases were excluded.

Digital multiplexed gene expression profiling:

Digital multiplexed gene expression profiling of patient samples was performed as previously described for all patients with sufficient yield and quality of RNA (N=10) with the nCounter® PanCancer IO360 Profiling Panel and the nCounter® Human v3 miRNA Assay (NanoString) according to manufacturer’s protocol [1,5]. Normalization was performed by nSolver analysis software with default settings (subtracting the mean + 2 standard deviations of negative control as a cut-off and adjustment for geometric mean of defined positive controls and defined housekeeper genes).

As a reference cohort for PSEL-FL, we used unpublished targeted DNA sequencing data from the GALLIUM cohort that enrolled patients with symptomatic, advanced stage classic FL (cFL-AS) [6-7]. An additional cohort of limited stage classic FL (cFL-LS) was kindly provided by Heike Horn (Dr. Margarete Fischer-Bosch-Institute of Clinical Pharmacology, Stuttgart, Germany) (N=43).

Data analysis and statistics:

RStudio software was used to generate Oncoplots and gene mutation summaries (package ggplots2, maftools). Gene expression data was normalized and analyzed for differential expression with the nSolver analysis software (version 4.0) by default parameters. Ratios (log2 fold changes) and normalized counts were exported for visualization, clustering, and gene set enrichment analysis with RStudio software (packages stats, ComplexHeatmap, EnhancedVolcano, ClusterProfiler). Global significance scores were calculated using nSolver nCounter advanced analysis software (version 2.0). Targeted mutational burden (TMB) was calculated as the mean of all total counts of non-silent variants at a VAF > 10% per sample as previously described [8]. Only genes that were captured in both cohorts (cFL-AS and cFL-LS) by targeted gene sequencing were used for calculation. TMB frequencies between cohorts were compared using ordinary one-way ANOVA followed by Tukey´s multiple comparisons test by GraphPad Prism Software (version 9). Results are represented as mean ± standard deviation (SD).

**Supplemental References**

1. Bararia D, Hildebrand JA, Stolz S et al. Cathepsin S Alterations Induce a Tumor-Promoting Immune Microenvironment in Follicular Lymphoma. *Cell Rep*. 2020 May 5;31(5):107522. DOI: https://doi.org/10.1016/j.celrep.2020.107522.
2. Luoma EK, Raininko R, Nummi PJ et al. Suitability of cerebrospinal fluid as a signal-intensity reference on MRI: evaluation of signal-intensity variations in the lumbosacral dural sac. *Neuroradiology*. 1997 Oct;39(10):728-32. DOI: https://doi.org/10.1007/s002340050496
3. Pastore A, Jurinovic V, Kridel R et al. Integration of gene mutations in risk prognostication for patients receiving first-line immunochemotherapy for follicular lymphoma: a retrospective analysis of a prospective clinical trial and validation in a population-based registry. *Lancet Oncol*. 2015 Sep;16(9):1111-1122. DOI: https://doi.org/10.1016/S1470-2045(15)00169-2
4. Li H, Durbin R. Fast and accurate short read alignment with Burrows-Wheeler transform. *Bioinformatics*. 2009 Jul 15;25(14):1754-60. DOI: https://doi.org/10.1093/bioinformatics/btp324
5. Hellmuth JC, Louissaint A Jr, Szczepanowski M et al. Duodenal-type and nodal follicular lymphomas differ by their immune microenvironment rather than their mutation profiles. *Blood*. 2018 Oct 18;132(16):1695-1702. DOI: https://doi.org/10.1182/blood-2018-03-837252
6. Marcus R, Davies A, Ando K et al. Obinutuzumab for the First-Line Treatment of Follicular Lymphoma. *N Engl J Med*. 2017 Oct 5;377(14):1331-1344. DOI: https://doi.org/10.1056/NEJMoa1614598
7. Jurinovic V, Passerini V, Oestergaard M et al. Evaluation of the m7-FLIPI in Patients with Follicular Lymphoma Treated within the Gallium Trial: EZH2 mutation Status May be a Predictive Marker for Differential Efficacy of Chemotherapy. *Blood*. 2019; 134 (Supplement_1): 122. DOI: https://doi.org/ 10.1182/blood-2019-130208
8. Alig S, Jurinovic V, Pastore A er al. Impact of age on genetics and treatment efficacy in follicular lymphoma. *Haematologica*. 2018 Aug;103(8):e364-e367. DOI: https://doi.org/10.3324/haematol.2018.187773

**Figure Legend**

**Fig. S1**: **a** Signal intensity of tumor masses compared to CSF in patients with aggressive PSEL (N=4) compared to patients with indolent PSEL (N=3). **b** Mutation frequency of single genes in the PSEL cohort compared to reported mutations frequencies of nodal B-NHL (de Leval, Blood 2022). **c** Mutation frequency of single genes in the PSEL-FL cohort compared to mutations frequencies in the cFL-LS and cFL-AS cohort. **d** Multiplex immunfluorescence of PSEL-FL and cFL (CD20, VEGFR2, TGFß, phospho-MAPK).

**Table Legend**

**Table S1**: Summarized clinical data of the PSEL cohort.

**Table S2:** Targeted gene sequencing of the PSEL cohort with non-silent variant count matrix and mutational calls.

**Table S3:** Differential gene expression analysis. Log2 fold change of analyzed genes (IO360) in the PSEL cohort compared to cFL-LS cohort (all genes and top 20 differentially expressed genes).
