# Supplementary figures and images for: Clinical and molecular features of primary spinal epidural lymphomas
Source: Ann Hematol. 2025 Sep 25;104(10):5493–9. doi: 10.1007/s00277-025-06554-0 (PMC12619745; doi:10.1007/s00277-025-06554-0)

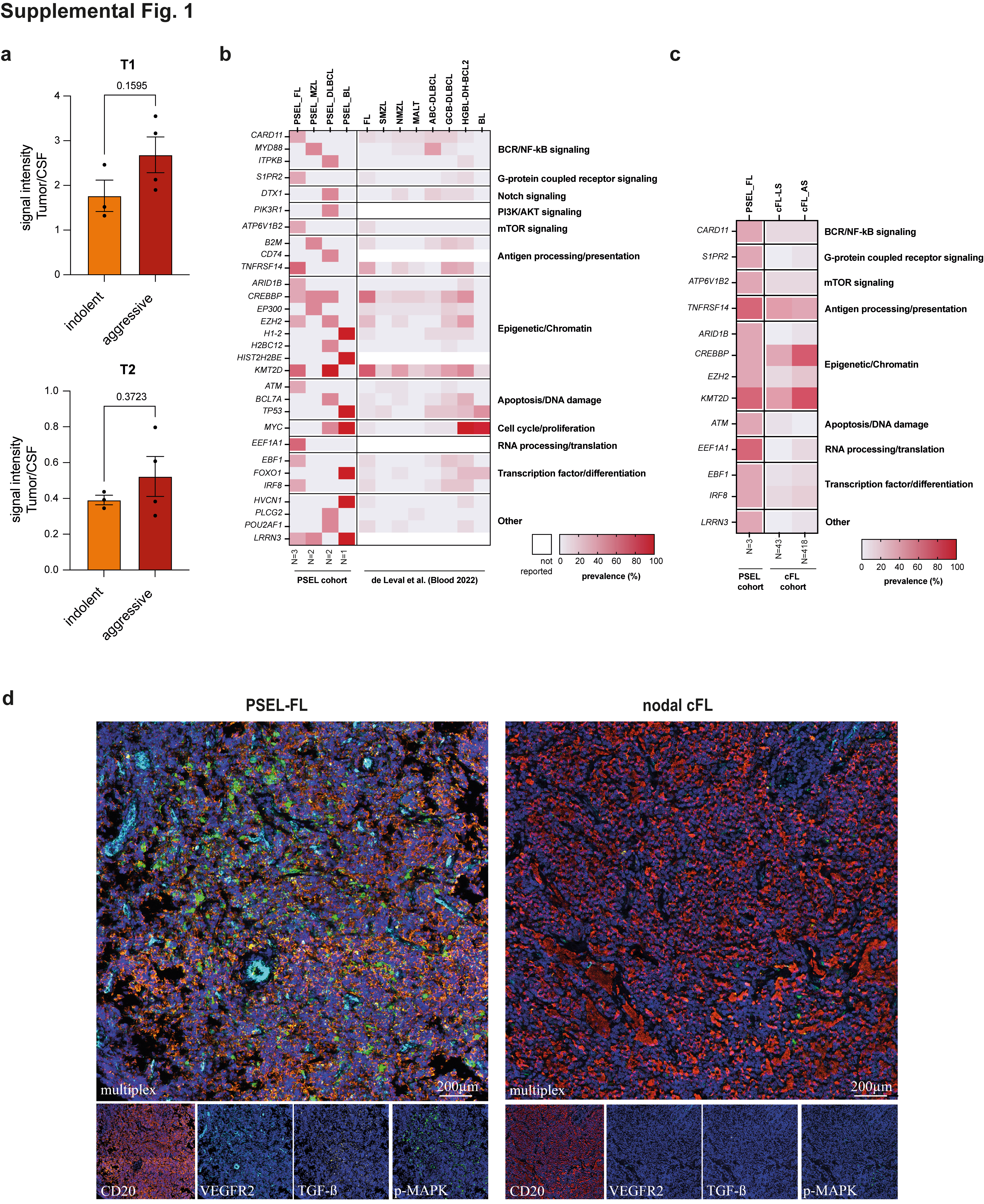

Supplement: Supplementary file 2 — (PNG 17.8 MB) [file 277_2025_6554_Fig3_ESM.png]
